# Antioxidant properties of polyphenols from snow chrysanthemum (*Coreopsis tinctoria*) and the modulation on intestinal microflora *in vitro*

**DOI:** 10.1080/13880209.2022.2117386

**Published:** 2022-09-10

**Authors:** Minghao Zhang, Naiyu Zhao, Minhao Xie, Deqiao Dong, Weilin Chen, Yuanpeng He, Dalin Yan, Haiyan Fu, Xinlin Liang, Li Zhou

**Affiliations:** a National Demonstration Center for Experimental Ethnopharmacology Education, School of Pharmaceutical Sciences, South-Central MinZu University, Wuhan, P. R. China; b Collaborative Innovation Center for Modern Grain Circulation and Safety, and College of Food Science and Engineering, Nanjing University of Finance and Economics, Nanjing, P. R. China; c Medscience-Tech Institute for Non-communicable Diseases at Optics Valley, Wuhan, P. R. China

**Keywords:** Phenolic compounds, intestinal microbiota, UPLC-QE Orbitrap/MS, prebiotic effect

## Abstract

**Context:**

*Coreopsis tinctoria* Nutt (Asteraceae), named snow chrysanthemum, is known to have a high level of polyphenols. However, the potential prebiotic effect on modulating intestinal microflora is still unclear.

**Objective:**

The chemical composition, antioxidant properties of snow chrysanthemum polyphenols (SCPs) and their effects on human intestinal microbiota were investigated.

**Materials and methods:**

SCPs were extracted using ultrasonic-assisted extraction, and further determined using UPLC-QE Orbitrap/MS. Five assays were used to investigate the antioxidant activities of SCPs. Subsequently, the effects of SCPs on intestinal microbiota *in vitro* were determined by high throughput sequencing and bioinformatics analysis.

**Results:**

Marein, isookanin and cymaroside were the major phenolic compounds, which accounted for 42.17%, 19.53% and 12.25%, respectively. Marein exhibited higher scavenging capacities in DPPH (EC_50_ = 8.84 µg/mL) and super anion radical assay (EC_50_ = 282.1 µg/mL) compared to cymaroside and isookanin. The antioxidant capacity of cymaroside was weakest among the three phenolic compounds due to the highest EC_50_ values, especially for superoxide anion radical assay, EC_50_ > 800 µg/mL. The result of *in vitro* fermentation showed that the three phenolic compounds increased the relative abundances of *Escherichia/Shigella*, *Enterococcus*, *Klebsiella*, etc., and isookanin notably increased the relative abundance of *Bifidobacterium* and *Lactobacillus*.

**Discussion and conclusions:**

SCPs exhibited antioxidant properties and potential prebiotic effects on modulating the gut microbiota composition. The findings indicated that SCPs consumption could exert prebiotic activity that is beneficial for human health.

## Introduction

*Coreopsis tinctoria* Nutt (Asteraceae), originated in North America and is now distributed worldwide (Guo et al. 2015). *Coreopsis tinctoria*, an annual herb, possesses important medicinal and economic value. *Coreopsis tinctoria* is mainly distributed in coastal states of North America, introduced into China around 1900, commonly known as ‘snow chrysanthemum’, which is widely cultivated in the Kunlun Mountain (3000 m above sea level) in the Xinjiang Uygur Autonomous Region of China. The flowers of this plant are widely used as traditional medicines due to the high levels of flavonoids, phenolic compounds, and terpenoids. These bioactive substances endow *C. tinctoria* with multiple pharmacological activities, especially antioxidant activity is the most important, which is affected by the chemical structures of polyphenols (Chen et al. 2016; Jing et al. 2016; Ma et al. 2016; Li et al. 2019). Few literature references were available for the effects of ultrasonic-assisted extraction on their chemical characteristics and antioxidant properties. Hence, it is necessary to identify and quantify phenolic compounds of snow chrysanthemum (SCPs) and evaluate their antioxidant properties.

*Coreopsis tinctoria* contains high levels of polyphenols, which have several biological activities including anti-inflammatory, antioxidant activities, anti-allergic and anticancer (Yang et al. 2016). However, polyphenols are poorly absorbed in the small intestine and approximately 90 ∼ 95% of ingested polyphenols can pass to the colon, where they regulate the structure of intestinal flora, particularly increasing certain health-promoting gut microbiota, such as *Lactobacillus* and *Bifidobacterium*. Polyphenol intake can increase the ratio of *Bacteroidetes* to *Firmicutes*, which contributes to the regulation of lipid metabolism (Wang et al. 2019). The gut microbiota breaks down and utilizes indigestible polyphenols to generate a series of micromolecular metabolites. Short-chain fatty acids (SCFAs) are the main metabolites during the fermentation of polyphenols, which have an important effect on the balance of gut microbiota growth and function. Furthermore, SCFAs are connected with energy metabolism, inflammation, immunoreaction and epithelium proliferation (Chai et al. 2012); the best known SCFAs are acetate, propionate, and butyrate (Tuohy et al. 2012). Therefore, gut microbiota plays a critical role in the decomposition and metabolism of polyphenols. Numerous *in vitro* and *in vivo* shreds of evidence support the regulative impact of plant polyphenols on gut microbiota. For instance, blueberry anthocyanins could promote the growth of the relative abundance of *Bifidobacterium* spp. (Zhou et al. 2020). The grape polyphenols significantly promote the growth of *Bifidobacterium* spp. and *Lactobacillus-Enterococcus* group and suppress the growth of *Clostridium histolyticum* group and *Bacteroides*-*Prevotella* group (Zhou et al. 2016). Consumption of polyphenol-rich kiwifruit by healthy rats for 4 weeks could significantly increase the abundance of *Lactobacillus* and *Barnesiella* and inhibit *Enterococcus*, *Escherichia* and *Staphylococcus* (Alim et al. 2020). Herein, plant polyphenols play an important role in reshaping the gastrointestinal tract and strengthening the host-microbial interactions with human health.

The current studies of *C. tinctoria* mainly focus on extraction technology, chemical compositions and bioactivity (Zălaru et al. 2014; Guo et al. 2015; Yao et al. 2016). To our best knowledge, no study was carried out on the effects of SCPs on the intestinal microbiota. Moreover, the beneficial effects of polyphenols on gut microbiota vary based on their chemical structures, glycation pattern, and hydroxyl groups. Different polyphenols have different impacts on gut microbiota. It is thereby meaningful to compare the fermentation mechanisms of different polyphenols from *C. tinctoria*.

In general, this work characterized the chemical structures of SCPs using UPLC-QE Orbitrap/MS, and their antioxidant activities were evaluated by five assays. Then, the effect of SCPs on the modulation of intestinal flora was investigated. This study could help consumers better understand the prebiotic functions of SCPs and promote the development and utilization of *C. tinctoria*.

## Materials and methods

The dried *C. tinctoria* flowers were purchased from a supermarket in November 2020, in Da Bancheng, Xinjiang Autonomous Regin, China. Voucher specimens of these samples were identified by the National Demonstration Centre for Experimental Ethnopharmacology Education, South-Central MinZu University Wuhan, China. The standards of marein, isookanin and cymaroside were purchased from Macklin Biochemical Technology Co., Ltd. (Shanghai, China). The Folin–Ciocalteu reagent was purchased from Aladdin Co. (Shanghai, China). All the solvents used for HPLC analyses were of HPLC grade, and those used for extraction were of analytical grade.

### SCP extraction

The dried flowers (5 g) were powdered and then soaked with 200 mL of 50% ethanol. The supernatant was separated after being centrifuged at 4000 *g* for 8 min. The substrate was re-extracted three times. Then, all the supernatant was collected and evaporated by a rotary evaporator (40 °C, 90 rpm). Finally, SCP extract was obtained and stored in a refrigerator at −18 °C with a relative humidity of 50 ± 5% for further use.

### Determination of total polyphenols

The determination of the total polyphenol content of SCP extract was performed by the Folin–Ciocalteu assay with some modification (Yu et al. 2002). The sample or standard solution (1 mg/mL, 50 μL), 250 μL of Folin–Ciocalteu reagent and 750 μL of 20% sodium carbonate were mixed well. The absorbance at 765 nm was tested after 2 h of incubation at ambient temperature using a Multimode Reader (TECAN, Männedorf, Switzerland). The results were reported as mg gallic acid equivalent/g dried *C. tinctoria* flower based on the calibration curve of gallic acid.

### Identification of polyphenols by UPLC-QE Orbitrap/MS

The phenolic compounds in SCPs were characterized by UPLC-QE Orbitrap/MS (LTQ-Orbitrap XL, Thermo Scientific). A TSKgel ODS-100Z column (150 mm × 4.6 mm, 5 μm, Tosoh) was applied for the analysis with a flow rate of 0.8 mL/min. The mobile phase was 0.1% aqueous formic acid (A) and acetonitrile (B). The gradient conditions used were as follows: 0–5 min, 90% A; 5–25 min, 90–60% A; 25–30 min, 60–40% A; 30–35 min, 40–90% A; 35–40 min, 90% A. The injection volume was 5 μL, and the detection wavelength was set at 285 nm, 378 nm. The detection was performed in positive mode with the data acquisition ranging from 50 to 600 Da. The collision energy was set at 25 V. The capillary temperature and auxiliary gas heater temperature were set at 325 °C and 350 °C, respectively.

The polyphenols were identified by comparing the retention time, high-resolution molecular ions and major MS^2^ fragment ions with those of the reference standards. The peak area of each molecular species was normalized relative to the total area.

### Purification of major phenolic compounds in SCP

The SCP extracts were further separated by an Agilent (Agilent Technologies, Inc., Santa Clara, CA, USA) semi-preparative chromatographic system with a DAD detector. Semi-preparative separation was achieved on a preparative Zorbax SB-C18 column (9.4 mm × 150 mm, Agilent, USA) with an isocratic elution of ACN-H_2_O (90:10, *v/v*). The autosampler was set to inject 50 μL SCP solution in methanol. Major phenolic compounds (marein, isookanin and cymaroside) were identified by comparison of their retention times with standard references and collected. The purity of each collected phenolic was examined by UPLC-QE Orbitrap/MS which is described before.

### Assay of DPPH free radical scavenging activity

Briefly, a DPPH reagent solution (0.1 mM) was freshly prepared in ethanol. SCP samples, including marein, isookanin and cymaroside were respectively dissolved in DPPH solution (3.125 ∼ 100 μg/mL). The solution was vortexed for 2 min and then incubated in the dark for 20 min at room temperature. The absorbance was read at 517 nm in a microplate reader (Tecan Spark 10 M, Switzerland). Ascorbic acid was used as a control. The DPPH radical scavenging activity was calculated with the following equation:

(1) $$\text{Scavenging activity }(\%)=[A_{0}-(A_{1}-A_{2})]/A_{0}\times 100\%$$

where *A*_0_ refers to control absorbance (DPPH in ethanol, 0.1 mM), *A*_1_ refers to sample absorbance, and *A*_2_ refers to blank absorbance ethanol.

### Assay of ferric reducing antioxidant power (FRAP)

FRAP assay was performed according to a method described by Benzie and Strain (1996) with slight modifications. FRAP reagent solution was freshly prepared by mixing 300 mM acetate buffer (pH 3.6), 10 mM 2,4,6-tripyridyl-s-triazine solution and 20 mM FeCl_3_·6H_2_O in a ratio of 10 *v*:1 *v*:1 *v*. 180 μL of FRAP working solution and 5 μL of standard solution or sample were mixed well. The mixed solution was incubated in the dark for 5 min. The absorbance was detected at 593 nm. Ascorbic acid was used as a control. Aqueous solutions of Fe^2+^ at concentrations of 0.03 ∼ 1 mg/mL were used for calibration, and the results were expressed as mM Fe^2+^.

### Assay of ABTS^•+^ radical scavenging activity

The 2,2′-azino*bis*(3-ethylbenzothiazoline-6-sulphonic acid) (ABTS^•+^) radical scavenging activity was evaluated according to the method described by Re et al. (1999). The ABTS^•+^ solution was prepared by mixing 7 mM ABTS^•+^ with 0.1 M phosphate buffered saline solution in a ratio of (1:1). The mixed solution was placed in the darkroom for 10–14 h, and then diluted to an absorbance of 0.7 at 734 nm. Then, 100 μL of the resulting ABTS^•+^ solution was mixed with 10 μL of each polyphenol sample solution, and the absorbance was measured at 734 nm. The ABTS^•+^ radical scavenging effect was calculated in the same equation described in the DPPH assay.

### Assay of superoxide anion radical scavenging activity

The superoxide anion radical scavenging was tested according to a previous study with some modifications (Bi et al. 2013). 50 μL of sample solution (25 ∼ 800 μg/mL), 50 μL NBT solution (78 μM), 100 μL NADH solution (78 μM) and 100 μL PMS solution (10 μM) were mixed well. Then, the mixture solution was vortexed and incubated at 25 °C for 5 min. The absorbance was read at 560 nm. The radical scavenging activity was calculated as follows:

(2) $$\text{Superoxide anion radical scavenging activity }(\%)=[A_{0}-(A_{1}-A_{2})]/A_{0}\times 100\%$$

where *A*_0_ refers to control absorbance, *A*_1_ refers to sample absorbance, and *A*_2_ refers to blank absorbance (phosphate buffer instead of NBT solution)

### Assay of reducing powder

Sample solution (2.5 mL) was added to 1% potassium ferricyanide (2.5 mL). The mixture was kept at room temperature (22–25 °C) for 20 min. Afterwards, 10% trichloroacetic acid (2.0 mL) was added to the stock solution and centrifuged (3000 *g*) for 15 min. The supernatant was taken and mixed with 0.5 mL of ferric chloride (0.1%, *w/v*). The absorbance was read at 700 nm. Ascorbic acid was used as control and the results were evaluated by subtracting the sample absorbance with blank absorbance.

### *In vitro* faecal fermentation

*In vitro* fermentation method was slightly modified according to the procedure described by Bai et al. (2021). Briefly, fresh human faeces were taken from 8 healthy donors (4 males and 4 females, 20–26 years old). All donors were without any antibiotic exposure for at least 6 months. The volunteers were mentally and physically able to participate in the study and informed written consent was obtained from each volunteer. In addition, the experiments were performed in compliance with the relevant laws and institutional guidelines. Fresh faecal samples were collected in anaerobic tubes and then dissolved in the physiological saline solution (NaCl 9.0 g/L, cysteine-HCl 0.5 g/L) to yield 10% (*w/v*) faecal slurry suspension after centrifugation. Autoclaved basal nutrient growth medium was supplied with *C. tinctoria* phenolics or nothing. Medium (18 mL) and 2 mL faecal slurry were added into the anaerobic tubes, then placed in the 37 °C anaerobic chambers. At each time point of fermentation (0, 12 and 24 h), assigned anaerobic tubes were taken out and stored at −80 °C for further analysis.

### Extraction of faecal DNA and 16S sequencing

Extraction of the total bacterial genomic DNA from all the faecal matter was performed with a commercial TIANamp Stool DNA Kit (TIANGEN Biotech Co., Ltd., Beijing, China). The V3-V4 hypervariable regions of the 16S rRNA gene from gut microbiota were amplified using universal primers. The MiSeq system (Illumina, San Diego, California, USA) was applied to sequence the extracted samples. High-quality reads were classified into operational Taxonomic Units (OTUs) at a 97% similarity threshold using the RDP database (Cole et al. 2014). Rarefaction curves and Shannon index were performed using the MOTHUR software (Schloss et al. 2009). Principal component analysis (PCA) was performed to provide an overview of gut microbial dynamics. Non-metric multidimensional scaling (NMDS) and Bray–Curtis similarity clustering analysis were performed using R software (version 2.15.3, http://www.r-project.org/) with a vegan package. Linear discriminant analysis effect size (LEfSe) analysis (http://huttenhower.sph.harvard.edu/galaxy/) was carried out to discover biomarkers to characterize the differences among three groups (Segata et al. 2011).

### Statistical analysis

All experiments were conducted on triplicate samples. The differences among groups were assessed by a one-way analysis of variance (ANOVA) procedure followed by Tukey’s test with SPSS software version 19.0 (Chicago, IL, USA). The statistical significance was set at *p* < 0.05.

## Results

### SCP content

The total polyphenol content of 50% ethanol SCP extract was 63.1 mg GAE/g determined in this study. The result was higher than that of 1.06–12.72 mg GAE/g in the hot-H_2_O extracts and 5.31–35.60 mg GAE/g in the 75% methanol extracts from 17 chrysanthemums including *C. tinctoria* (Li et al. 2019). Taking together, these results demonstrated that *C. tinctoria* might significantly differ in their TPC due to the different extraction methods.

### Characterization of polyphenols in SCP extract

**Figure 1.**
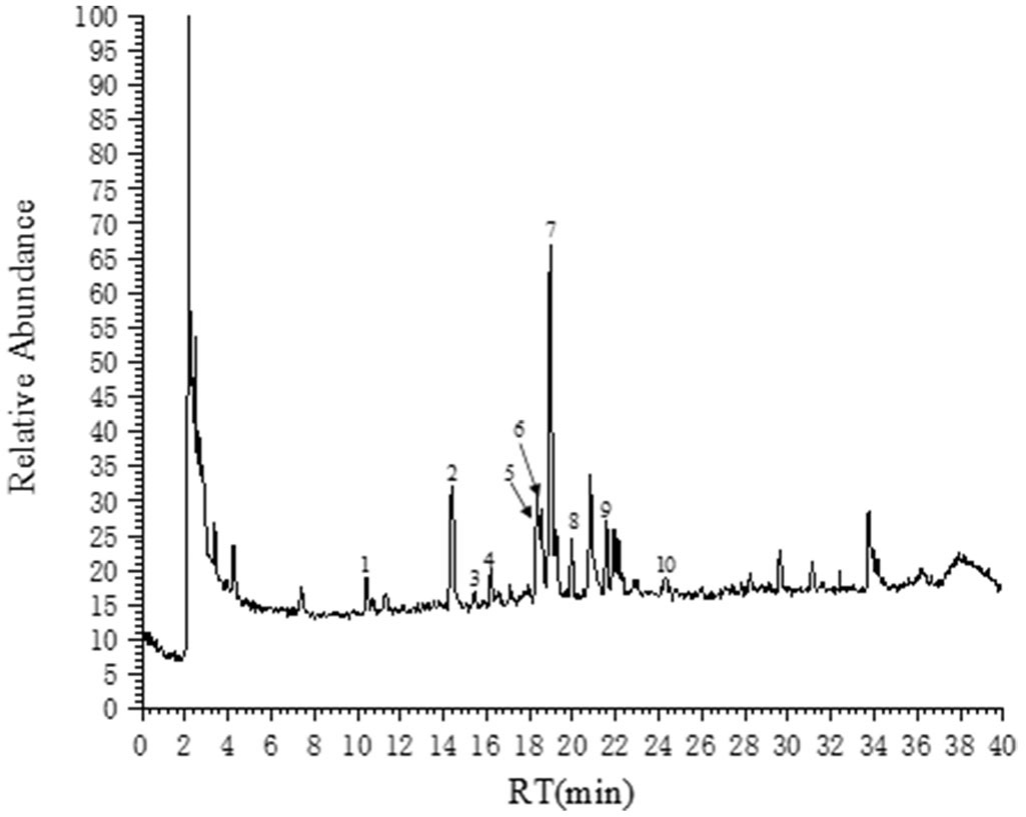
Chromatogram of polyphenol identified in *C. tinctoria* extracts using UPLC-QE Orbitrap/MS.

A total of 10 polyphenols in SCP extracts were detected by UPLC-QE Orbitrap/MS analysis (Figure 1). Amongst, three predominant phenolic compounds were marein (peak 7), isookanin (peak 2) and cymaroside (peak 5), which accounted for 42.17%, 19.53% and 12.25%, respectively (Table 1). Other peaks were not polyphenols which were shown in Supplementary Figure S1.

**Table 1.** Molecular species profiles of polyphenols from SCP.

Peak	*R_t_* (min)	Formula	[M + H]^+^			Structure	%
			Experimental mass (*m/z*)	Theoretical mass (*m/z*)	Mass error (ppm)		
1	10.43	C_21_H_22_O_12_	467.11586	467.11840	−5.438	Taxifolin-7-*O*-β-D-glucoside	2.31
2	14.40	C_15_H_12_O_6_	289.07042	289.07066	−0.830	Isookanin	19.53
3	15.39	C_21_H_22_O_10_	435.12866	435.12857	0.207	Flavanocorepsin	1.81
4	16.22	C_21_H_20_O_13_	481.09756	481.09767	−0.229	Quercetagitin-7-*O*-glucoside	2.11
5	18.38	C_21_H_20_O_11_	449.10767	449.10784	−0.379	Cynaroside	12.25
6	18.58	C_15_H_12_O_6_	289.07047	289.07066	−0.657	Okanin	8.97
7	18.96	C_21_H_22_O_11_	451.12329	451.12349	−0.443	Marein	42.17
8	19.98	C_15_H_12_O_7_	305.06528	305.06558	−0.983	Taxifolin	5.01
9	21.55	C_21_H_22_O_10_	435.12857	435.12857	0	Coreopsin	4.73
10	24.28	C_15_H_12_O_6_	289.07053	289.07066	−0.450	5,7,3′,5′-Tetrahydroxy-flavanone	1.11

Further validation was tested by the standards of marine, isookanin and cymaroside. the phenolic compounds were quantified on the basis of corresponding external standard curves. The linear regression coefficients (*R*^2^) of all the lipid standards were above 0.990. Meanwhile, the LOD and LOQ values were less than 0.8 and 1.3 ng/mL, respectively. The accuracies of lipid profiles were in the range of 90–105.

**Figure 2.**
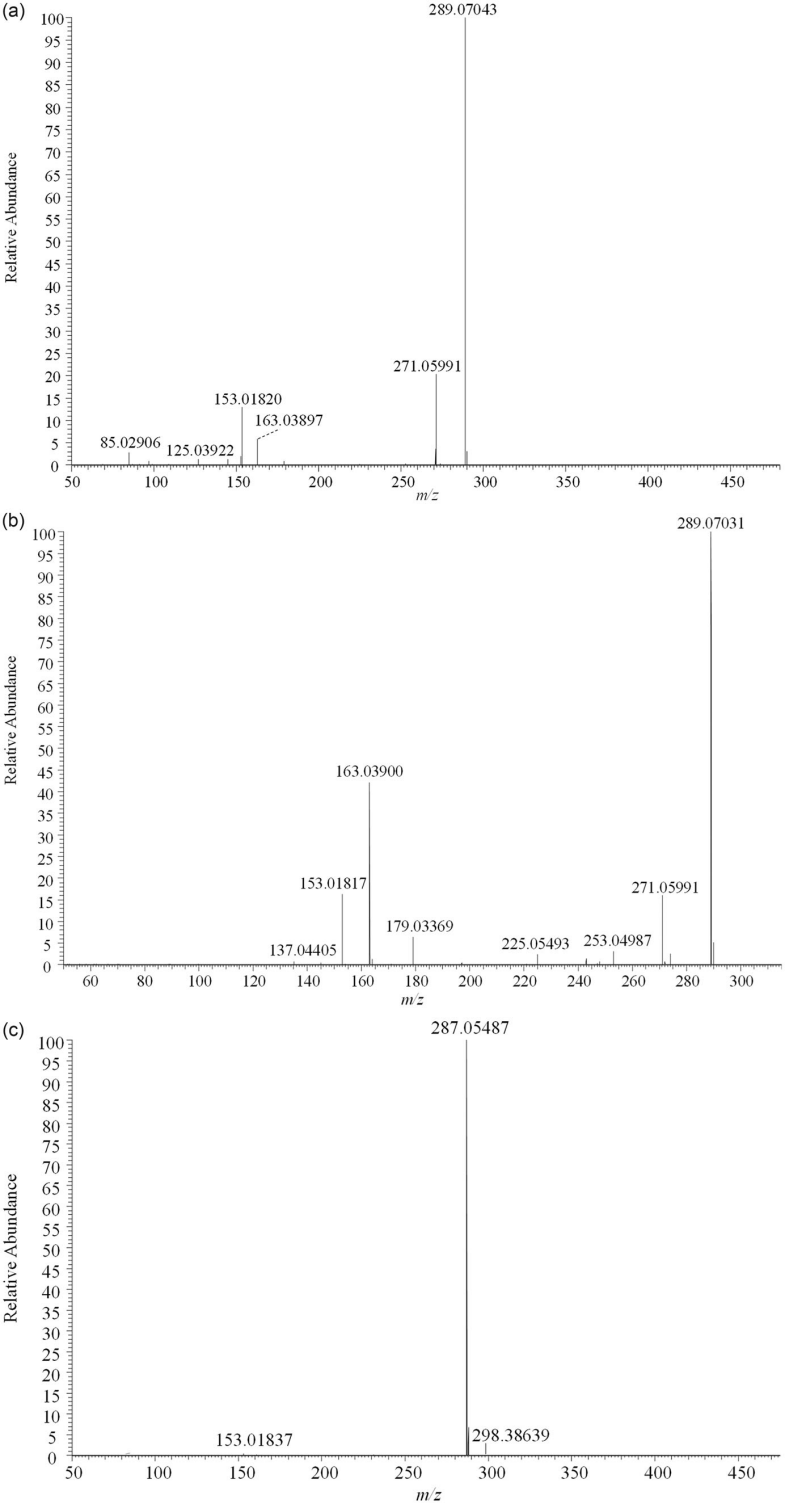
MS/MS fragmentation pathway of (a) marein (*m/z* 451.12329), (b) isookanin (*m/z* 289.07031) and (c) cymaroside (*m/z* 449.10532) under positive ion mode.

Figure 2 shows the fragmentation pathway of the three major polyphenols. Peak 6 had a molecular ion ([M + H]^+^) at *m/z* 451.12329, and the major fragments at 289.07043 ([M + H-glucose]^+^), 271.05991 ([M + H-glucose-H_2_O]^+^), 153.01820 ([M + H-glucose-H_2_O-C_8_H_6_O]^+^), 125.03922 ([M + H-glucose-H_2_O-C_8_H_6_O-CO]^+^), suggesting that the molecular structure was marein (Figure 2(a)). As for peak 2, it had an *m/z* signal of 289.07031. The fragment ions at *m/z* 271.05991, corresponded to the loss of H_2_O. *m/z* 153.01817 corresponded to the residual fragment of [M + H-glucose-H_2_O-C_8_H_6_O]^+^, and *m/z* 137.04405 referred to the fragment of [M + H-glucose-H_2_O-C_8_H_6_O-O]^+^ (Figure 2(b)). Herein, peak 2 was identified as isookanin (Li et al. 2019). Peak 4 was tentatively identified as cymaroside (*m/z* 449.10532), which contained a fragment ion at *m/z* 287.05487. The result was in conformity with the residual fragment of the loss of caffeoyl ([M + H-caffeoyl]^+^) (Chen et al. 2016; Figure 2(c)).

### Antioxidant properties of polyphenols

**Figure 3.**
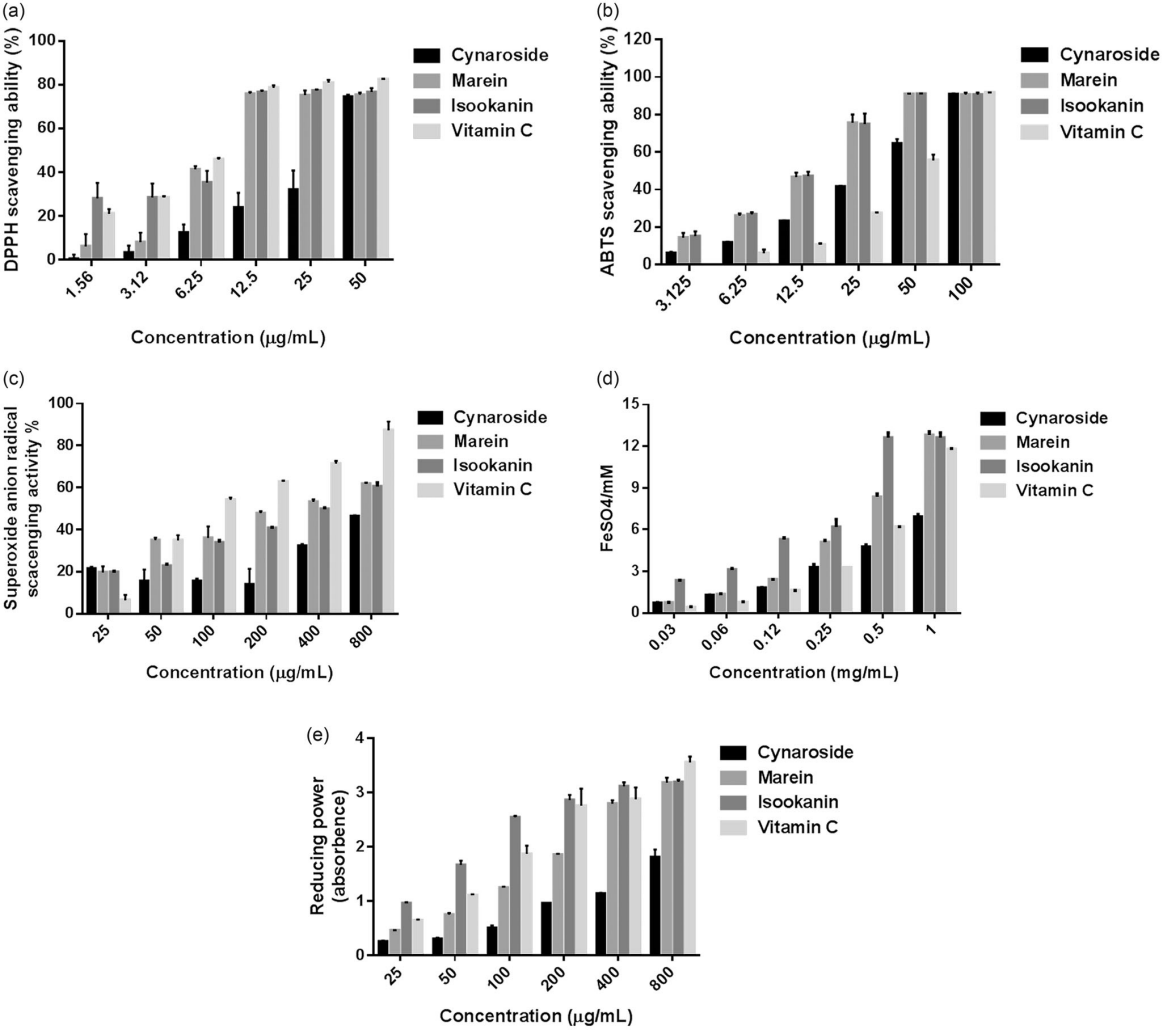
Scavenging effects on DPPH radical (a), ABTS radical (b), superoxide radical (c), FRAP (d) and reducing power (e).

Figure 3 shows that the major polyphenols (cymaroside, marein and isookanin) possessed antioxidant capacities, and exhibited concentration dependence. According to the variation curves, the scavenging abilities of the three polyphenols on ABTS^•+^ radical and FRAP exhibited significantly higher than *Vc*. As for DPPH and reducing power, their scavenging abilities were higher than *Vc* in low concentration but gradually equal to *Vc* with the increase of reagent concentration. In contrast, the superoxide anion radical scavenging of SCPs showed a highly concentration-dependent increase although their antioxidant abilities were lower than *Vc*. It manifested that SCPs exhibited a greater capability to contribute hydrogen to superoxide anion because of the weaker dissociation energy of the O–H bond (Xie et al. 2017).

The antioxidant effects of the samples were evaluated and compared by the low concentration capable of inhibiting 50% of the radical (EC_50_). A lower EC_50_ value means that a lower sample concentration was needed to eliminate 50% of the free radicals. The EC_50_ values of DPPH radical scavenging activity, ABTS^•+^ radical scavenging activity and superoxide anion radical scavenging activity were given in Table 2. In ABTS^•+^ assay, scavenging ability by SCP and *Vc* was higher than that in DPPH assay. Additionally, marein exhibited higher scavenging capacities in DPPH and super anion radical assay compared to cymaroside and isookanin, while isookanin showed higher scavenging capacities in FRAP and reducing power assay. Notably, the antioxidant capacity of cymaroside was significantly weakest (*p* < 0.05) among the three phenolic compounds.

**Table 2.** DPPH, FRAP, ABTS˙^+^ and superoxide anion radical scavenging of SCP.

Variety	EC_50_		
	DPPH	ABTS˙^+^	Superoxide anion
Ascorbic acid	7.97^a^	41.05^c^	115.1^a^
Cynaroside	30.81^b^	29.89^b^	>800^c^
Marein	8.84^a^	12.61^a^	282.1^b^
Isookanin	7.08^a^	12.47^a^	383.5^bc^

Different letters in the same row indicate significant difference at *p* < 0.05 by Duncan’s test.

### Effects of SCPs on intestinal microbiota

**Figure 4.**
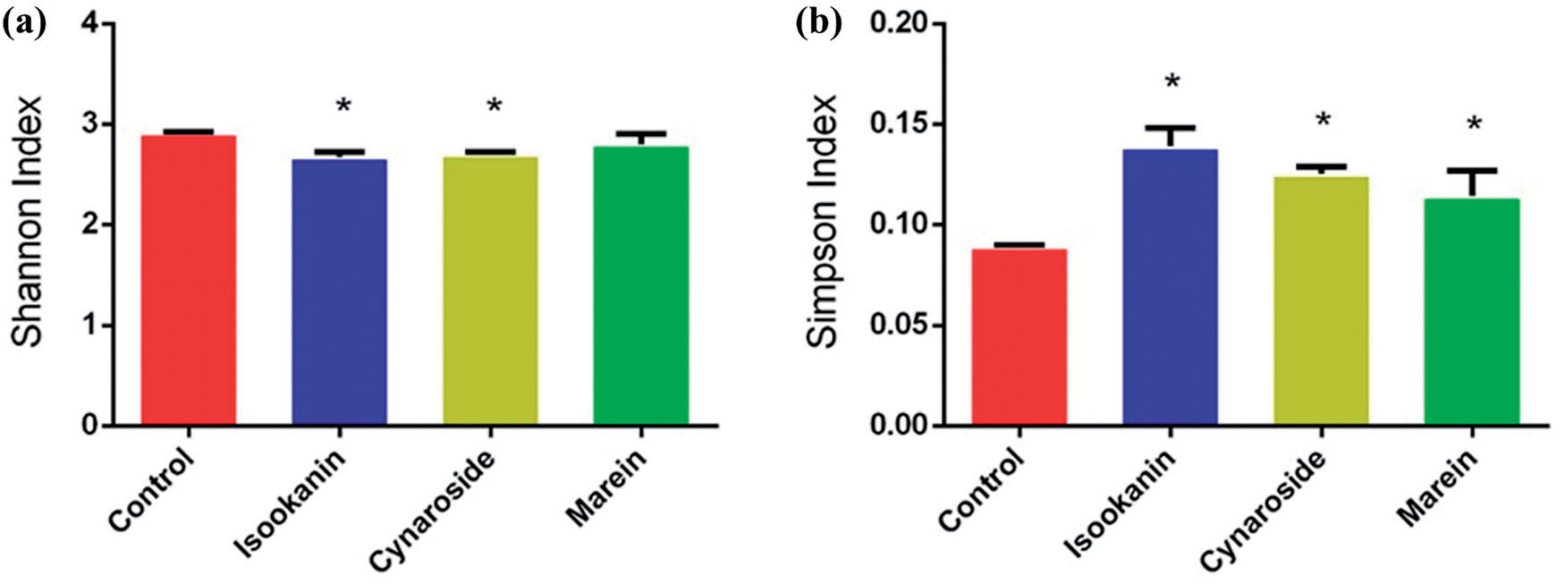
Shannon index (a) and Simpson index (b) of control and *C. tinctoria* phenolics treated groups.

**Figure 5.**
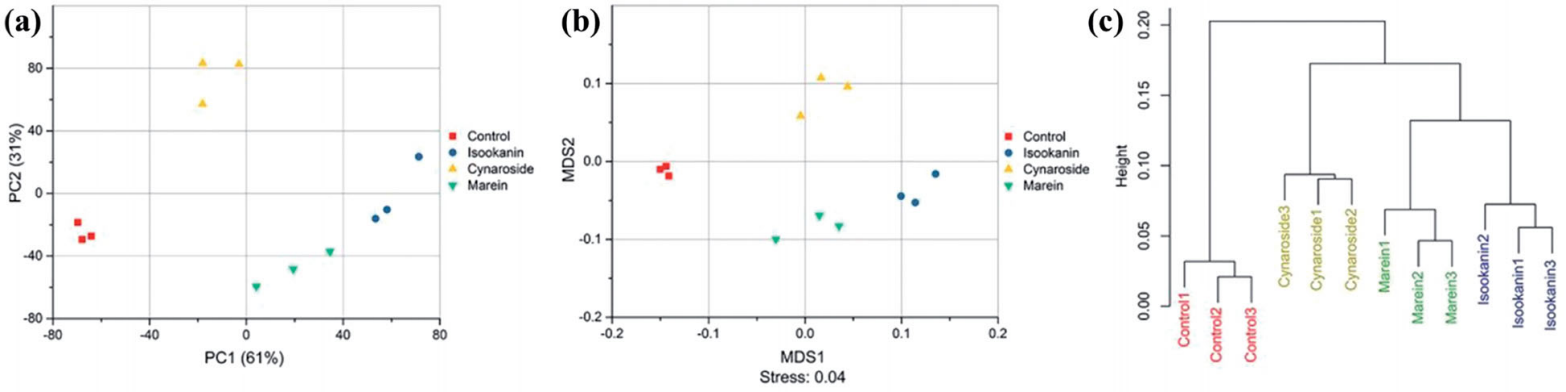
PCA (a), NMDS (b) and cluster analysis (c) of microflora at OTU level.

**Figure 6.**
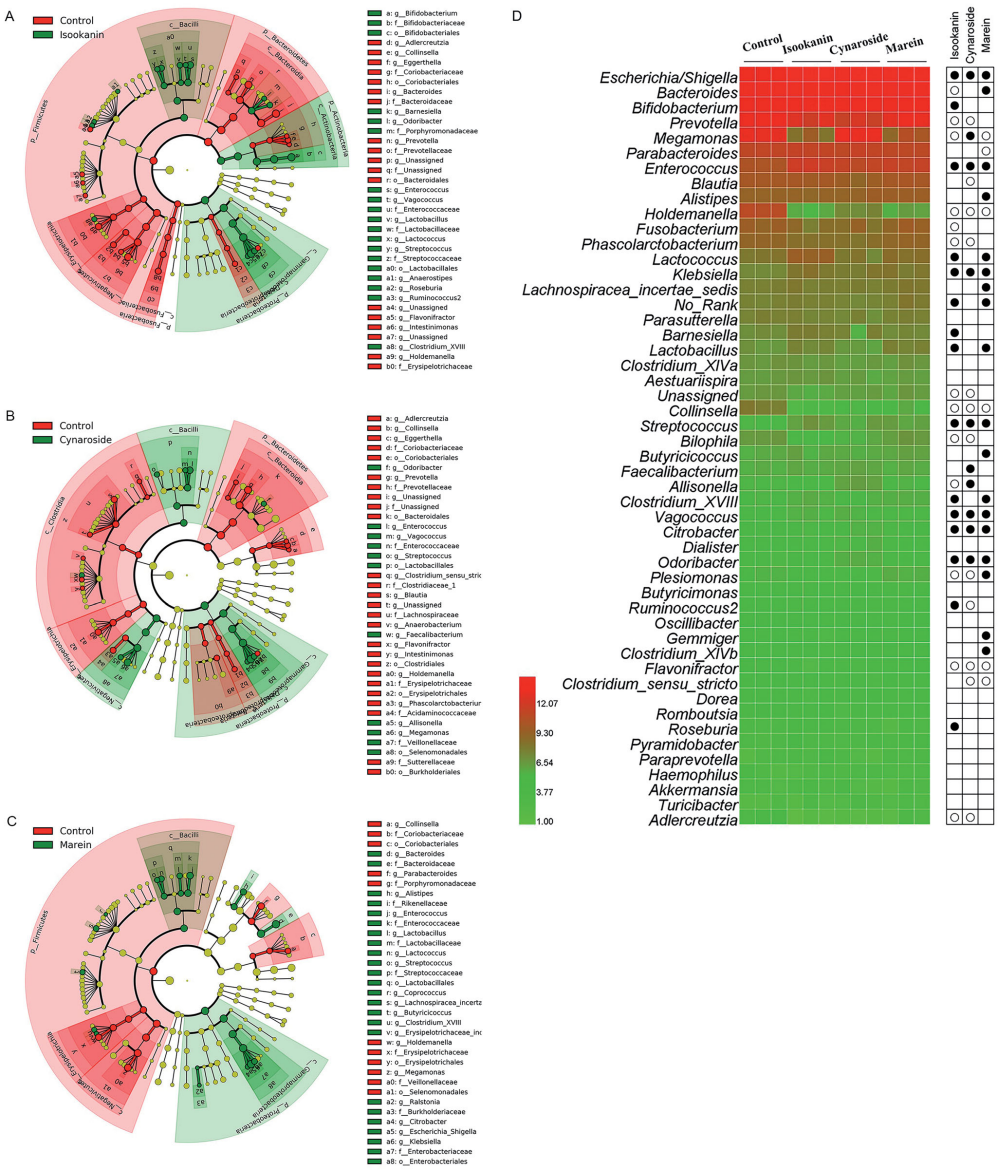
Cladograms of distinct taxons derived from isookanin (A), cynaroside (B), and marein (C), heatmap and LDA of microbial compositions at genus level (D). (● means that the relative abundance of this genus was increased by the phenolic treatment than control; ○ means the relative abundance of this genus was reduced than control).

Isookanin and cynaroside had significant effects on both Shannon and Simpson index (*p* < 0.05), and marein only increased Simpson index (Figure 4). The similarities of microbial profiles were compared by PCA, NMDS and cluster analysis (Figure 5). The different taxa between the control and treated groups were identified by LEfSe, and the results were displayed in Figure 6. The three phenolic compounds showed some similar and distinct effects on modulating the microbial communities. Isookanin increased the relative abundances of Actinobacteria and Proteobacteria at the phylum level, and Actinobacteria, Bacilli and Gammaproteobacteria at the class level, and decreased the relative abundances of Firmicutes, Fusobacteria and Bacteroidetes, Fusobacteriiae, Negativicutes, Erysipelotrichia, Deltaproteobacteria and Bacteroidia. Cynaroside increased the relative abundances of the class of Bacilli, Negativicutes, and Gammaproteobacteria, and decreased the relative abundances of Clostridia, Erysipelotrichia, Deltaproteobacteria and Bacteroidia. Marin increased the relative abundances of Bacilli and Gammaproteobacteria and decreased the relative abundances of Erysipelotrichia and Negativicutes. All the three phenolic compounds could increase the relative abundances of *Escherichia/Shigella*, *Enterococcus*, *Klebsiella*, *Streptococcus*, *Vagococcus*, *Citrobacter*, and *Odoribacter* at the genus level. Noteworthy that only isookanin increased the relative abundance of *Bifidobacterium*, *Barnesiella*, and *Roseburia*. Isookanin and cynaroside exhibited a contrary effect on the relative abundance of *Megamonas*, *Allisonella*, and *Ruminococcus2*. The relative abundance of *Lactobacillus* was enhanced by isookanin and marein, but *Akkermansia* was not affected by the three phenolics.

**Figure 7.**
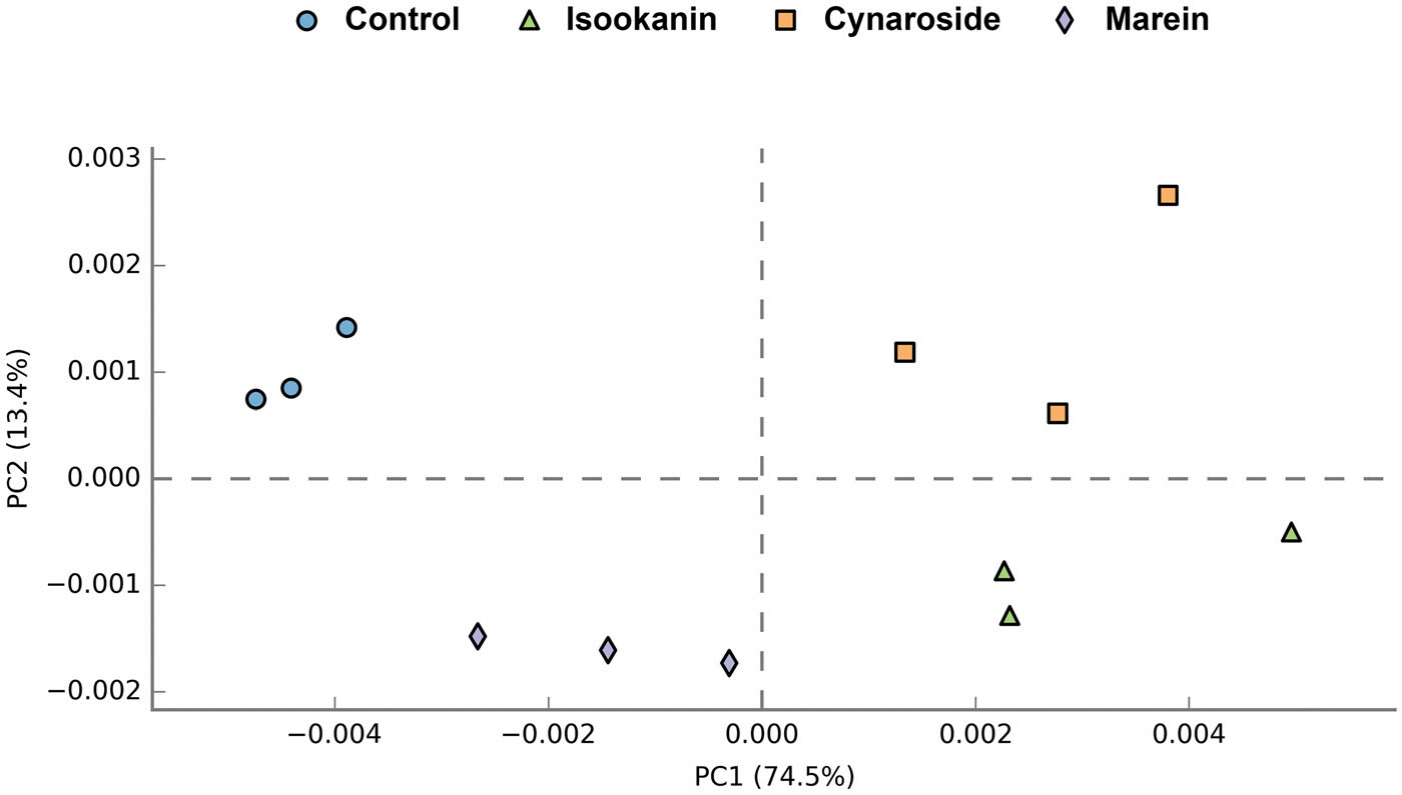
PCA of functional analysis of microbiota.

**Figure 8.**
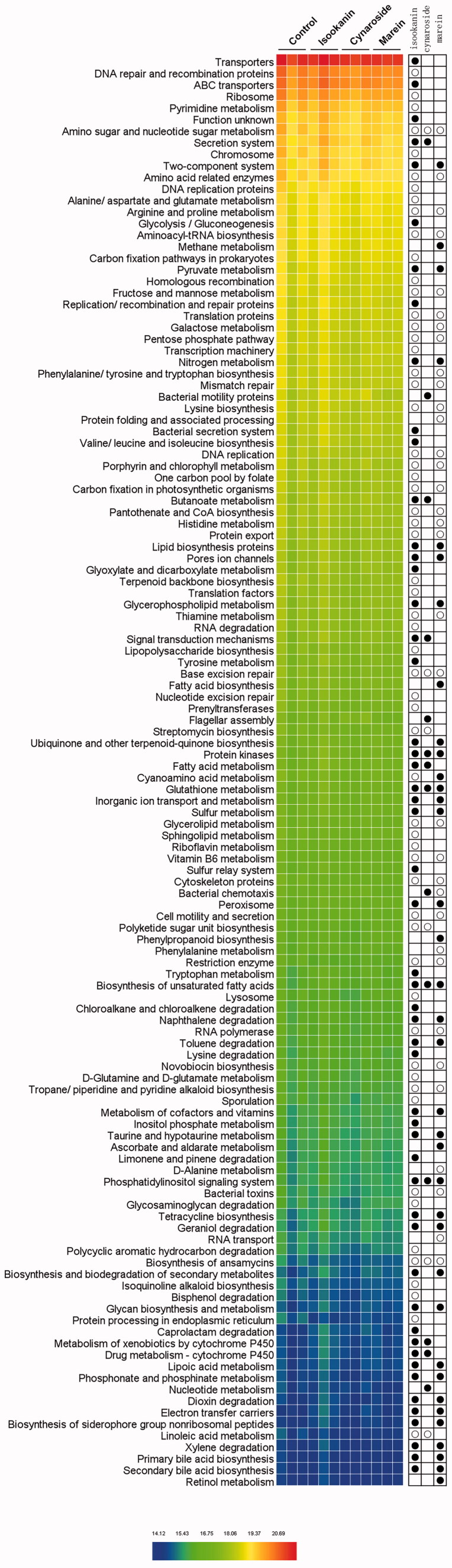
The effects of *C. tinctoria* phenolics on microbial functions predicted by PICRUSt. ● indicates this functional pathway was up-regulated by the present phenolic compound compared to control, and ○ means it was down-regulated.

Besides the taxonomic structures of the microbial profiles, the functions of the microbial communities were also predicted and analyzed by PICRUSt. As shown in Figure 7, the PCA based on functional profiles revealed that the control group was completely separated from the phenolics treated groups, and isookanin had the most significant impact, showing a similar tendency with their taxonomic compositions. The three phenolic supplements upregulated the KEGG pathways of protein kinases, glutathione metabolism, biosynthesis of unsaturated fatty acids, and phosphatidylinositol signalling system, and downregulated the amino sugar and nucleotide sugar metabolism, base excision repair, and biosynthesis of ansamycins (Figure 8).

## Discussion

The three major phenolic compounds, marein, isookanin and cymaroside, were chosen and subsequently isolated from SCP extract. The purity was shown to be at least 97.3% for each phenolic compound. Then, they were further used to investigate the antioxidant activity and the effect on human intestinal microflora. Five assays, such as DPPH free radical scavenging activity, FRAP, ABTS^•+^ radical scavenging activity, superoxide anion radical scavenging activity and reducing powder, were used to investigate the antioxidant activities of SCPs. In ABTS^•+^ assay, scavenging ability by SCP and *Vc* was higher than that in DPPH assay. The reason might be that ABTS^•+^ assay is more applicable to examine hydrophilic antioxidants while DPPH assay is more suitable to examine hydrophobic antioxidants (Floegel et al. 2011). The three polyphenols (cymaroside, marein and isookanin) were hydrophobic polyphenols, which were slightly soluble in an aqueous solution, but easily soluble in ethanol. Therefore, the scavenging ability of SCP examined by the DPPH assay was stronger than ABTS^•+^ assay. Our results were in agreement with a previous study, which described that the scavenging ability of the anthocyanins from *Lycium ruthenicum* Murr (Solanaceae) was more favourable against liposoluble free radicals (DPPH) than that against water soluble free radicals (ABTS^•+^) (Wang et al. 2018).

As polyphenols have been regarded as prebiotics (Gibson et al. 2017), the effects of the three phenolic compounds, including isookanin, cynaroside, and marein, isolated from *C. tinctoria* on intestinal microecology *in vitro* were tested by anaerobic fermentation. The alpha diversities indicate the richness and evenness of microbial community in samples, and they were significantly affected by SCP. Several *in vitro* and *in vivo* studies have demonstrated the prebiotic effects of polyphenols, including the inhibition of the growth of *Fusobacterium* spp., *Clostridium* spp., *Pseudomonas* spp., *Salmonella* spp., *Bacillus* spp., *Escherichia coli*, *Helicobacter pylori*, and the increase of the beneficial groups, such as *Lactobacillus* spp., *Bifidobacterium* spp., *Akkermansia* spp. and *Faecalobacterium* spp (Cueva et al. 2020). The improvements in *Gammaproteobacteria*, *Gemellaceae*, *Turicibacter*, *Atopobium*, *Lachnospira*, *Anaerostipes*, *Akkermansia*, *Roseburia*, *Prevotella*, *Bacteroides*, *Enterococcus*, *Prevotella*, *Blautia*, *Eubacterium*, and *Eggerthella*, and reduction in *Bacteroides*, *Rikenellaceae*, *Ruminococcus*, *Oscillospira*, *Clostridium*, *Alistipes*, *Oribacterium*, *Odoribacter*, and *Butyricimonas* have also been reported in previous studies about the effects of other polyphenols or phenolic-rich foods on microbiota (Moorthy et al. 2020). Phenolics could modulate microbiota by selectively inhibiting the growth of certain microbial species or communities due to the toxicity of the phenolic groups (Espin et al. 2017). They could interfere with cell membrane functions and bacterial energy metabolism, reduce biofilm formation and expression of virulence factors and pathogenic genes, and bind to proteins and enterotoxins (Espin et al. 2017). In the present study, it was found that the SCP, especially isookanin, could reduce the primary metabolism functions of the microbiota *in vitro*, indicating its selective suppressing effects on the microflora. Our results manifested that the *in vitro* microecology modulated the effects of isookanin, cynaroside, and marein, as well as their contributions to the functions of the microbial communities. They could exhibit a different impact from other phenolics, and provide the potential to be in the arsenal for personalized nourishment or medicine. In addition, our results showed that the microbial profiles of the three components supplied samples were significantly shifted from the control samples, and isookanin contributed to the largest effect on microbiota. The data indicated that all the isookanin, cymaroside and marein significantly changed the overall microbial community structures.

Furthermore, phenolics had effects on several metabolism pathways. At least two kinds of the compounds increased the metabolism of pyruvate, nitrogen, butanoate, glycerophospholipid, fatty acid, inorganic ion, cofactors and vitamins, taurine and hypotaurine and xenobiotics of the microbiota, and decreased the metabolism of carbohydrates (fructose, mannose and galactose metabolism, pentose phosphate pathway), amino acids (amino acid related enzymes, aminoacyl-tRNA biosynthesis, translation proteins, histidine metabolism, phenylalanine, tyrosine, tryptophan and lysine biosynthesis), lipids (linoleic acid and glycerolipid metabolism), cofactors (vitamin B_6_ and thiamine metabolism, pantothenate and CoA biosynthesis), nucleic acid (RNA polymerase, DNA replication). Notably, isookanin and marein increased primary and secondary bile acids biosynthesis, biosynthesis and biodegradation of secondary metabolites and decreased the bacterial toxins. Isookanin also decreased lipopolysaccharide biosynthesis.

## Conclusions

SCPs were obtained by ultrasonic-assisted extraction. Ten phenolic compounds were identified by UPLC-QE Orbitrap/MS. Amongst them, marein, isookanin and cymaroside, were the major phenolics in SCPs. The antioxidant capacity of cymaroside was significantly weakest (*p* < 0.05) compared with marein and isookanin. The three polyphenols had significant effects on the intestinal microbial profiles and functions of the communities. They increased the relative abundances of *Escherichia/Shigella*, *Enterococcus*, *Klebsiella*, *Streptococcus*, *Vagococcus*, *Citrobacter*, and *Odoribacter* and isookanin notably increased the relative abundance of *Bifidobacterium* and *Lactobacillus*. The phenolics also had an impact on several metabolism pathways. This study helps consumers better understand the nutritional value of *C. tinctoria*, and further provides a scientific basis for the development of *C. tinctoria* and its products.

## Supplementary Material

Supplemental Material
