# Supplementary material for: Antioxidant properties of polyphenols from snow chrysanthemum (Coreopsis tinctoria) and the modulation on intestinal microflora in vitro
Source: Pharm Biol. 2022 Sep 10;60(1):1771–80. doi: 10.1080/13880209.2022.2117386 (PMC9467560; doi:10.1080/13880209.2022.2117386)
Supplement: Supplemental Material [file IPHB_A_2117386_SM1552.docx]

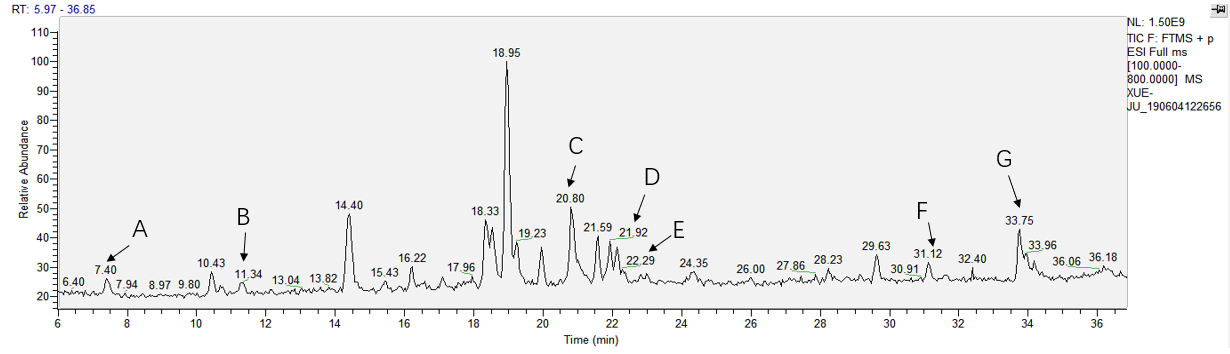


**Figure S1.** Chromatogram of non-polyphenol identified in *C. tinctoria* extracts using UPLC-QE Orbitrap/MS. **A**, desethyl atrazine; **B**, chlorogenic acid; **C**, *m/z* 701.4932 unknown; **D**, *m/z* 396.8015 unknown; **E**, *m/z* 234.2064 unknown **F**, (1R,5S)-3-(3-(5,9-dimethyl-7-oxo-3-phenyl-7H-furo[3,2-g]chromen-6-yl)propanoyl)-3,4,5,6-tetrahydro-1H-1,5-methanopyrido[1,2-a][1,5]diazocin-8(2H)-one;**G**, (1'S,2'S,3S,3'S,7'R,8'R,9'S,

13'R)-8'-(acetyloxy)-2,2,2',9',13'-pentamethyl-6',16'-dimethylidene-6,11',15'-trioxo-10',14',17'-trioxaspiro[oxane-3,5'-pentacycloheptadecane]-3'-yl (2E)-2-methylbut

-2-enoate; H, isopalmitic acid.
